# Sex Differences in Opioid Response Linked to *OPRM1* and *COMT* genes DNA Methylation/Genotypes Changes in Patients with Chronic Pain

**DOI:** 10.3390/jcm12103449

**Published:** 2023-05-13

**Authors:** Laura Agulló, Javier Muriel, César Margarit, Mónica Escorial, Diana Garcia, María José Herrero, David Hervás, Juan Sandoval, Ana M. Peiró

**Affiliations:** 1 Pharmacogenetic Unit, Alicante Institute for Health and Biomedical Research (ISABIAL), Dr. Balmis General University Hospital, Pintor Baeza, 12, 03010 Alicante, Spain; 2 Clinical Pharmacology, Toxicology and Chemical Safety Unit, Institute of Bioengineering, Miguel Hernández University, Avda. de la Universidad s/n, 03202 Elche, Spain; 3 Pain Unit, Department of Health of Alicante, Dr. Balmis General University Hospital, c/Pintor Baeza, 12, 03010 Alicante, Spain; 4 Epigenomics Core Facility, La Fe Health Research Institute, Ave. Fernando Abril Martorell, 106, 46026 Valencia, Spain; 5 Pharmacogenetics Unit, La Fe Health Research Institute, Ave. Fernando Abril Martorell, 106, 46026 Valencia, Spain; 6 Department of Applied Statistics and Operations Research and Quality, Universitat Politècnica de Valéncia, 46022 Valencia, Spain

**Keywords:** sex differences, chronic pain, epigenetics, DNA methylation, pharmacogenetics

## Abstract

Analgesic-response variability in chronic noncancer pain (CNCP) has been reported due to several biological and environmental factors. This study was undertaken to explore sex differences linked to *OPRM1* and *COMT* DNA methylation changes and genetic variants in analgesic response. A retrospective study with 250 real-world CNCP outpatients was performed in which data from demographic, clinical, and pharmacological variables were collected. DNA methylation levels (CpG island) were evaluated by pyrosequencing, and their interaction with the *OPRM1* (A118G) and *COMT* (G472A) gene polymorphisms was studied. A priori-planned statistical analyses were conducted to compare responses between females and males. Sex-differential *OPRM1* DNA methylation was observed to be linked to lower opioid use disorder (OUD) cases for females (*p* = 0.006). Patients with lower *OPRM1* DNA methylation and the presence of the mutant G-allele reduced opioid dose requirements (*p* = 0.001), equal for both sexes. Moreover, *COMT* DNA methylation levels were negatively related to pain relief (*p* = 0.020), quality of life (*p* = 0.046), and some adverse events (probability > 90%) such as constipation, insomnia, or nervousness. Females were, significantly, 5 years older with high anxiety levels and a different side-effects distribution than males. The analyses demonstrated significant differences between females and males related to *OPRM1* signalling efficiency and OUD, with a genetic–epigenetic interaction in opioid requirements. These findings support the importance of sex as a biological variable to be factored into chronic pain-management studies.

## 1. Introduction

Some inherent biological differences contribute to sex differences in chronic noncancer pain (CNCP) [1,2], where females are more vulnerable to maintaining musculoskeletal pain with greater psychological distress [3,4]. Traditionally, it has been thought that such differences are largely due to the endogenous opioid system and hormonal regulation [5,6], but there are also genetic and epigenetic factors (i.e., DNA methylation, noncoding RNA expression, or histone modifications) [7,8] that could contribute as they do in other autoimmune disorders or neuropsychiatric diseases [9].

Current research suggests that there are significant differences between males and females in the genetics and epigenetics associated with chronic pain [10,11]. Some studies have identified specific genes and signalling pathways that are involved in pain sensation and perception [12], and these genes may be expressed differently in males and females [13]. In addition, epigenetics, which is the study of how environmental factors may influence gene expression, also appears to play an important role in sex differences in chronic pain [14].

In recent years, some genetic markers have been linked with interindividual differences in analgesic response [15,16], such as μ-opioid receptor 1 (*OPRM1*, A118G, rs1799971-G allele, 11–17% in the Caucasian population). This variant has been associated with higher doses of opioid requirements [17], and being more predisposed to compulsive behaviours and opioid dependence compared to rs1799971-A carriers [18,19]. In the same way, variants of the gene that encodes enzyme catechol-O-methyltransferase (*COMT*, G472A, and rs4680-A allele) are linked with a lesser capacity to metabolise monoamines and, thus, higher dopamine levels arise. Here, a lower pain threshold and increased vulnerability to chronic pain have been observed compared to the rs4680-G ancestral allele, and even more when combined with the *OPRM1* variant genotypes [20]. However, scientific evidence for the effect of these gene variants is not complete enough to explain the wide variability observed in the real world.

Hence, the possible involvement of a sex-mediated genetic–epigenetic interaction could be considered a modulator factor [21,22]. The aim of this study was to explore sex differences linked to DNA methylation/genotype changes that may affect the expression of the genes *OPRM1* and *COMT* by conditioning a different analgesic response.

## 2. Materials and Methods

### 2.1. Study Design

**Figure A1:**
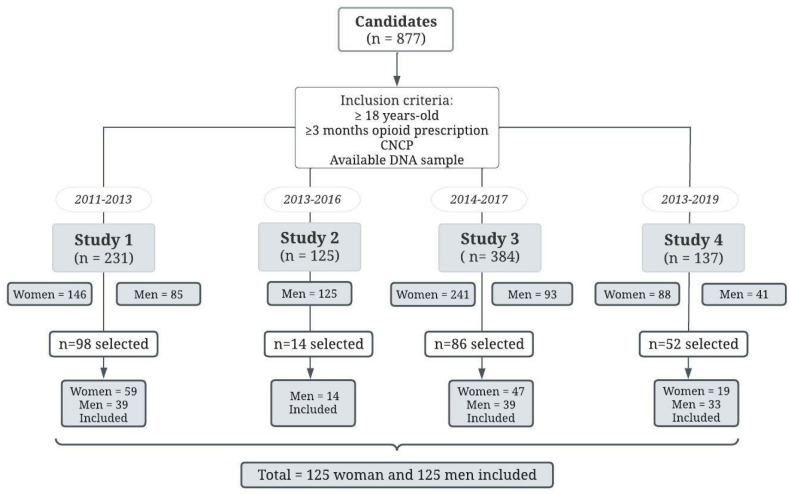
Flow chart of the included patients and their studies of provenance. CNCP, chronic noncancer pain.

A retrospective study (EPA-OD) was designed and conducted at the Pain Unit of the Alicante Health Department, Dr. Balmis General University Hospital, in Spain, from March 2021 to March 2022. The study was approved by the Ethics Committee (Protocol Code 2020-158). Written informed consent was waived due to the retrospective nature of the study. In any case, all the patients had already given informed consent to participate in previous observational studies done in the same setting [23,24]. The last study ended early due to the COVID-19 pandemic, as seen in Figure A1.

### 2.2. Participants and Data Collection

All the samples taken from the candidates in the present study (*n* = 250) were obtained from Biobank (Alicante Institute for Health and Biomedical Research (ISABIAL), Spain). This study adhered to the Spanish National Biobanks Network. Data were collected from original databases and completed from patients’ electronic health records. The inclusion criteria were patients aged ≥18 years old, CNCP (moderate or severe pain lasting at least 6 months) with long-term opioids (≥3 months), and with available DNA samples previously donated to Biobank. Patients under 18 years old, with oncologic pain or any psychiatric disorders that could interfere with the proper development of the study were excluded. Other chronic-pain syndromes of unclear pathophysiologies, such as fibromyalgia or neuropathic pain, such as painful polyneuropathy, postherpetic neuralgia, trigeminal neuralgia, and poststroke pain, were not included.

#### 2.2.1. Clinical Outcomes

A Global Pain State questionnaire [25], which qualitatively measures pain intensity and relief, was collected at the time that each patient was included in the study using the Visual Analogue Scale (VAS). This consists of a horizontal line ranging from 0 (lowest) to 100 mm (highest), where the patient points on the line the intensity of the pain or relief that he/she feels, respectively. Quality of life was evaluated through the EuroQol-5D-3L scale that consists of a VAS vertical line from 0 (the worst imaginable health status) to 100 mm (the best imaginable) where the patient indicates his/her actual health status. The patient’s diagnosis and demographic characteristics, such as age, sex, and employment status (active, retired, or work disability) were also registered. Psychological status was calculated with the Hospital Anxiety and Depression Scale: HADS, 0–21 scores, classified as normal (<7), probable (8–10) and case (>11 scores) [26].

#### 2.2.2. Pharmacology and Hospital Resources Use

Pharmacological variables such as the main opioid (i.e., tramadol, fentanyl, tapentadol, buprenorphine, oxycodone, and morphine) was registered (Table A2). In different opioid combinations, oral morphine equivalent daily dose (MEDD) was estimated using available references [27]; the number of adverse events was collected with a list of the most frequent analgesic side effects from the Summary of Product Characteristics frequency as “very common” or “common”, and a blank field to add any other adverse event was developed. Opioid use disorder (OUD) was diagnosed by a psychiatrist according to DSM-5 as part of an established opioid tapering procedure followed since 2018 [17].

### 2.3. Genetic/Epigenetic Data

At the time of enrolment in the original study, patient samples were collected for the pharmacogenetic analysis. Approximately 2 mL of saliva were collected in tubes containing 5 mL of PBS. Once the saliva sample was taken, it was stored at −80 °C until its processing. Genomic DNA was isolated using an E.N.Z.A. forensic DNA kit (Omega Bio-Tek Inc., Norcross, GA, USA) in accordance with the manufacturer’s instructions. In the present study, samples were provided by the Alicante BioBank and processed following standard operating procedures.

#### 2.3.1. Genotypes Analysis

The following gene variants were genotyped at the ISABIAL Molecular Biology Laboratory (Alicante GVA, Spain): *OPRM1* (rs1799971) and *COMT* (rs4680) using the realtime PCR rotor gene Q system (Qiagen, Hilden DE-NW, Germany), through the use of specific TaqMan MGB^®^ probes (Applied Biosystems, Pleasanton, CA, USA). Amplification parameters were as follows: pre-PCR for 10 min at 95 °C, 40 cycles for 15 s denaturation at 92 °C, and 1 min final extension at 60 °C.

#### 2.3.2. DNA Methylation Analysis

The Epigenomics Core Facility of the Health Research Institute La Fe performed the methylation analysis. Before this, a DNA integrity quality control was performed to ensure that DNA met standard quality measurements. All the DNA samples were assessed for purity using a NanoDrop 2000c (Thermo Fisher Scientific, Wilmington, DE, USA) with 260/280 and 260/230 ratio measurements and quantified by the fluorometric method (Quan-iT PicoGreen DsDNA Assay, Life Technologies, Carlsbad, CA, USA). Agarose gels at 1.5% were performed to assess DNA integrity. The obtained high-quality DNA samples (500 ng) were selected for bisulphite conversion using the EZ DNA Methylation kit (Zymo Research Corp., Irvine, CA, USA) following the manufacturer’s recommendations.

A triplet of primers was designed for each promoter region of genes *OPRM1* and *COMT* using Qiagen’s PyroMark Assay Design 2.0 software to hybridise to CpG-free sites to ensure methylation-independent amplification and pyrosequencing steps. Primers sequences are listed in Table A1 (all given as 5′ > 3′). Briefly, the PCR was performed under standard conditions with biotinylated primers. Pyrosequencing reactions and the DNA methylation quantification of *OPRM1* and *COMT* CpG sites located at their promoter regions were performed in a PyroMark Q24 System, version 2.0.7 (Qiagen), using appropriate reagents and recommended protocols. Samples were repeated if pyrosequencing runs did not pass the pyrosequencer quality checks or if the internal bisulphite conversion controls failed.

**Figure 1:**
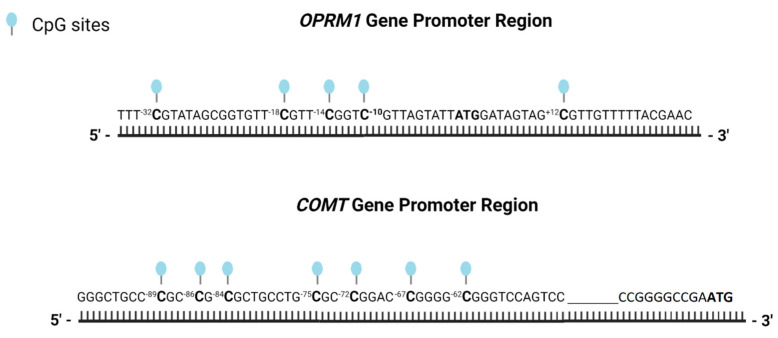
The *OPRM1* (μ-opioid receptor 1) and *COMT* (catechol-O-methyltransferase) gene promoter region. The locations of the CpG sites are represented by knobs and translation start sites (ATG) are shown in bold.

As shown in Figure 1, the CpG island we studied in the *OPRM1* gene (chr6: 154039512-154039571) is located between nucleotides −35 and +27 (relative to the adenine of the ATG translation start site). We examined five CpG dinucleotides located at nucleotides −32, −25, −18, −14, −10, and +12. The CpG dinucleotides −18 and −14 are located at a potential Sp1 binding site, and the CpG +12 site at a second binding site. The selection of these CpG sites was based on the previous study conducted by Nielsen et al. [28]. As for the *COMT* gene, seven CpG sites located between nucleotides −97 and −50 (chr22:19929354-19929398) of the MB-*COMT* promoter region were selected based on the work of Zhong et al. [29]. The MB-*COMT* promoter is part of a complex regulatory region that includes multiple enhancers and silencers that regulate the expression of the *COMT* gene.The seven specific CpG dinucleotides are located at nucleotides −89, −86, −84, −75, −72, −67, and −62.

### 2.4. Statistical Analysis

A convenient sample size of 250 participants (stratified by sex: 1:1 men/women) was defined due to the number of biological samples available at Biobank (ISABIAL, Spain). Data distribution was analysed by the Kolmogorov–Smirnov test following the Lilliefors correction method. A descriptive analysis of continuous quantitative variables (i.e., pain intensity, relief, and quality of life) was presented as the mean ± standard deviation (SD) while discrete variables (i.e., HADS scores and adverse events) are shown using their median and interquartile range (IQR). Categorical data (sex, employment status, anxiety and depression groups, and pharmacological prescription) were expressed by percentages (%).

The demographic, clinical, pharmacological, and epigenetic/genetic factors were compared using χ2 or Fisher’s exact test for the categorical variables, and the t-test or Mann–Whitney U test for the continuous variables depending on their distribution. When more than two groups were involved, ANOVA/Kruskal–Wallis or chi-square tests were used for continuous or categorical variables, respectively.

After performing the pyrosequencing technique, we obtained the methylation percentages of the *OPRM* and *COMT* genes. These values were used to carry out the analysis of the possible associations between the DNA methylation level and the selected variables, by means of a linear mixed-regression model using logarithmic transformation for the absolute values. A Bayesian regression analysis was also performed to analyse the association between DNA methylation and the presence of all the different adverse events. The probability of the effect of the variable being negative (higher methylation values, lower risk) or positive (higher methylation values, higher risk) is reported. An ordinal regression model was used to explain the DNA methylation-*OPRM1*/*COMT* genotypes interaction for clinical variables. Given the high correlation between the different methylation values of the CpG sites selected at the gene promoter region, only one CpG site per gene was selected to carry out the regression model (*COMT*-CpG6 and *OPRM1*-CpG2). Specifically, the selection of the CpG site was based on the degree of variability (the site with the highest variability was selected for each gene). Averaging the methylation values of the different CpGs of the region might introduce a bias since the average is not an observed variable. Nevertheless, the methylation values were so similar that the results would have been almost the same if including another CpG or even using the average in this case. The variable sex was included as a possible confounding factor. Statistical analyses were performed using the R software (v 4.0.3, Auckland, CA, USA). A *p* < 0.05 was considered to be statistically significant.

## 3. Results

A total of 250 candidates, 125 females and 125 males, were included after excluding patients who were duplicated between studies or did not meet the inclusion criteria. All included participants (Figure S1) were referred to our Pain Unit for routine pain management, mostly due to nonspecific low back pain (83%).

The sample’s mean age was 62 ± 14 years, 59% were retired, and all the participants were Caucasian residents of Spain. The mean for moderate pain intensity (67 ± 21 mm), pain relief (32 ± 27 mm), and quality of life (43 ± 23 mm) was equal for both sexes.

### 3.1. Sex Differences in the Demographic and Clinical Data

Females were a significant mean of 5 years older (64 ± 14 vs. 59 ± 14 years old, *p* < 0.05), have significantly higher nonspecific low back pain (95%, *p* < 0.001), significantly higher 4 anxiety scores (9 [5,13] vs. 5 [2,11] scores, *p* < 0.05), showed 15% more dry skin (31 vs. 16%, *p* < 0.05) and 17% more weight changes (33 vs. 16%, *p* < 0.05) compared to males. In contrast, males presented 9% higher OUD (26 vs.15%, *p* < 0.05) and 13% higher sexually adverse events (33 vs. 20%, *p* < 0.05) than females. Demographic and clinical data are shown in Table 1.

### 3.2. DNA Methylation/Genotypes and Analgesic Response

**Figure 2:**
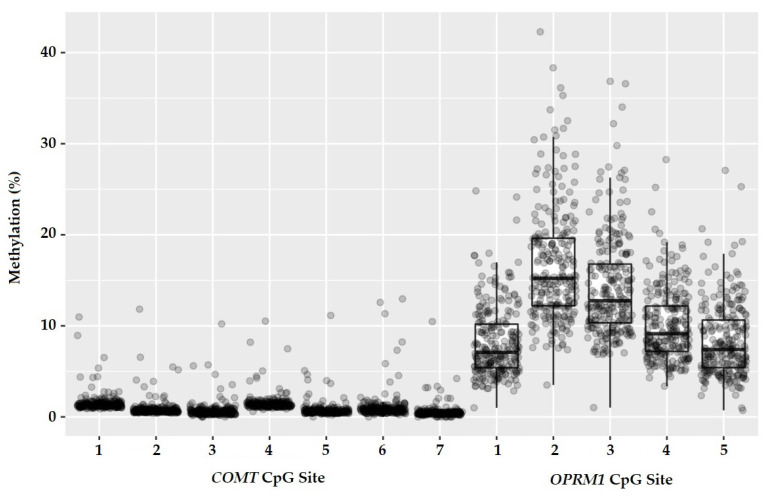
Distribution of methylation values (%) at each CpG site of the *COMT* (sites 1–7) and *OPRM1* (sites 1–5) genes.

The DNA methylation values obtained in the seven selected CpG sites of the *COMT* gene (sites 1–7) showed low variability, with values close to 0 (0.54–1.52%). However, the five selected CpG sites of the *OPRM1* gene (sites 1–5) were methylated to a larger extent with typical dynamic ranges between 8.2% and 16.6%. DNA methylation values at the selected CpG sites and the variability level appear in Table 2 and Figure 2, respectively.

**Figure A2:**
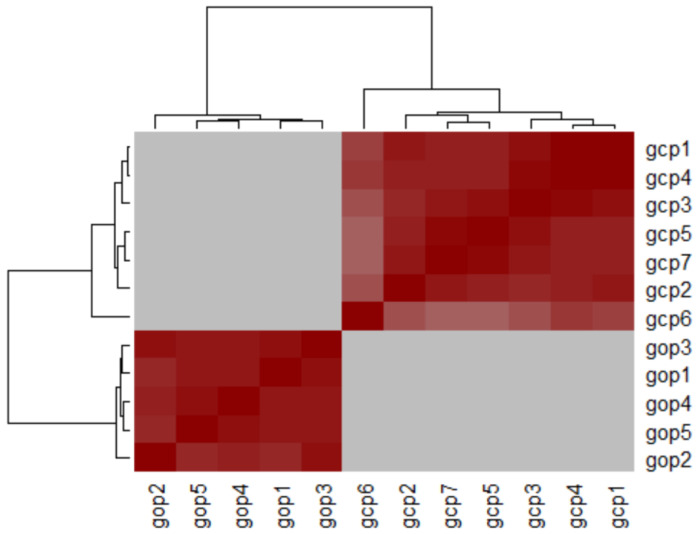
Degree of association between the methylation value of the different sites of OPRM1 (gene OPRM1 position, gop 1 to 5) and COMT (gene COMT position, gcp 1 to 7) genes. The intense garnet colours correspond to very high degrees of association.

As already mentioned in the statistical analysis section, the level of association between the different CpG sites located in each of the genes was high, and they were almost identical and provided hardly any additional information. The degree of association between the methylation value of the different CpG sites is depicted in Figure A2. Therefore, only one CpG site was selected from each gene (*COMT*-CpG6 and *OPRM1*-CpG2) and the percentages obtained were used to perform the regression analysis.

The obtained genotypic frequencies were equally distributed by sex in genes *OPRM1* (AA = 67; AG = 30; GG = 3%) and *COMT* (GG = 22; GA = 54; AA = 24%). Sex differences observed for the influence of *OPRM1* and *COMT* DNA methylation on clinical outcomes are shown in Table 3.

#### 3.2.1. Associations Linked to *OPRM1* DNA Methylation

**Figure 3:**
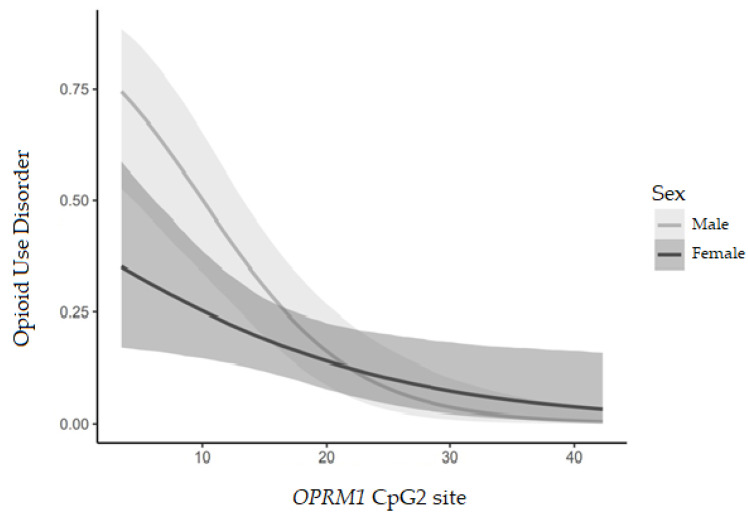
Effect of *OPRM1* DNA methylation (%) on opioid use disorder per sex according to male (M) or female (F).

Linear-regression models show that anxiety and OUD were negatively related to *OPRM1* DNA methylation levels (β = −0.178, *p* = 0.046 and β = −0.165 *p* < 0.001, respectively); furthermore, females had a lower OUD prevalence (β = −2.123, *p* = 0.006) but higher anxiety impact scores appeared (β = 1.869, *p* = 0.039). A sex interaction with *OPRM1* DNA methylation levels was observed due to OUD (β = 0.099, *p* = 0.047). Females with lower *OPRM1* DNA methylation levels presented fewer OUD prevalence than males, as shown in Figure 3.

Additionally, an ordinal regression model has been used to explain the association between *OPRM1* DNA methylation and genotype. The results show that the MEDD requirements were impacted by the *OPRM1*-G-allele (β = −0.914, *p* < 0.001), *OPRM1* DNA methylation (β = −0.023, *p* = 0.005), and their genotype/epigenetic interaction (β = 0.046, *p* = 0.001). The data suggest a MEDD reduction with the presence of mutant G-allele/lower *OPRM1* DNA methylation. In contrast, for higher *OPRM1* DNA methylation, no reducing effect of the G allele was observed, as shown in Table 3.

#### 3.2.2. Associations Linked to *COMT* DNA Methylation

The data show that when DNA *COMT* methylation increased, both pain relief (β = −3.15, *p* = 0.020) and quality of life (β = −2.07, *p* = 0.046) decreased. Furthermore, a positive correlation between pain relief and quality of life was found (Spearman r = 0.31, *p* < 0.001). Regarding the different adverse events, an inverse correlation of *COMT* was noted in relation to constipation, insomnia, dry mouth, dry skin, lack of appetite, red skin, and nervousness (probability > 90%). This means that a lower *COMT* DNA methylation level would imply a higher risk of these individual adverse events appearing. On the contrary, a positive correlation between *COMT* (the greater methylation, the higher the appearance risk) was observed for dizziness, as seen in Table 4.

## 4. Discussion

Our data showed significant sex differences related to *OPRM1* signalling efficiency in OUD, with an *OPRM1*-G allele interaction for the opioid dose requirement. A lower OUD probability appeared for females with decreased *OPRM1* DNA methylation. Additionally, sex conditioned a different anxiety level together with 5-years older females and a different side-effects pattern than males. These findings support the importance of sex as a biological variable to be factored into opioid management studies. Moreover, once data validation is performed, this information could be useful for developing predictive models of OUD based on sex and DNA methylation level, as well as for adjusting required opioid doses based on the genetic/epigenetic profile in clinical practice.

DNA methylation is a dynamic process that can change depending on different factors such as age, exposure to toxic substances, diet, and lifestyle. According to previous studies, a region of the genome is considered to be hypomethylated when the methylation level is less than 20%, while a region is considered to be hypermethylated when the methylation level is greater than 80% [30]. Both stages can affect gene expression and are related to various diseases and biological processes. However, these methylation thresholds are only a guide and should not be taken as absolute values to classify DNA methylation in all cases. It is important to keep in mind that reference values for normal DNA methylation should be considered in a broader context to understand its biological and clinical significance. For this reason, in this study, we have studied the associations between DNA methylation level and clinical, pharmacological and safety variables, but we have not categorized the methylation values obtained.

### 4.1. OPRM1 DNA Methylation and Opioid Use Disorder

Epigenetic mechanisms provide a platform that represents the convergence between the combined effect of biological and environmental influences on sex differences. However, data must be carefully interpreted for making gene-regulation predictions, which can vary in life spans based on DNA methylation changes at a few CpG sites. Conversely to our results, the literature shows that a lower *OPRM1* gene expression may condition higher OUD rates in patients with long-term opioid use, such as cancer-pain patients [31], subjects in methadone programmes [32], or former heroin addicts [28]. An increase in DNA methylation of CpG sites in the *OPRM1* promoter may block the binding of Sp1 and other transcription factors, which can reduce protein and mRNA expression and final *OPRM1* silencing [33]. New hypotheses arise about the possibility of a sex difference DNA methylation pattern in patients as a consequence of long-term opioid use history and/or of the presence of OUD. For potent drugs such as opioids, initial exposure is a crucial phase on the path to dependence and addiction, and it is reasonable to expect some epigenome modifications to occur during the first few exposures [34]. The question is whether epigenetic changes are induced after repeated opioid exposures or if, on the contrary, these are indicators of early epigenomic and potentially transcriptomic responses. This should be profoundly explored together with sex differences in the methylation pattern.

Similarly, the limited but growing literature based on human studies has demonstrated that DNA methylation changes occur in response to environmental stress or lifestyle factors, such as physical activity. Exercise is a commonly prescribed treatment for chronic low-back pain, and sex-specific epigenetic mRNA gene expression adaptation, in response to endurance exercise, has been reported. Yet it is uncertain why global DNA methylation after exercise is similar between males and females despite the difference in mRNA expression of the epigenetic regulatory genes [35]. This may support the notion that dysregulated histone acetylation can be an important mechanism for memories of life stress that occurred early in life and can increase visceral pain in adulthood [1,36] or different gene networks function in the peripheral nervous systems that may contribute to sex differences in pain with rats after nerve injury [37]. Understanding the underlying biological mechanism of this different health risk may help to shed light on a possible sexually dimorphic risk for, or resilience from, developing OUD [38]. Therefore, although we have described the potential role of DNA methylation in OUD prevalence, further research is needed to unravel the role of the interaction among the different epigenetic factors in this regulatory context.

### 4.2. OPRM1 Methylation-Genotype Interaction in MEDD

Our data indicated that *OPRM* 118-G allele carriers were associated with a lower requirement of MEDD to achieve analgesia. Previous data suggest that the presence of homozygous ancestral-natural-type AA alleles of SNP *OPRM1* (A118G/dbSNP rs1799971-G) protects against pain perception and reduces problems that derive from pain perception, which preserves mobility, improves self-care, reduces anxiety-related problems in patients, and diminishes activities of daily living-related problems. Conversely to our results, patients who are G-allele carriers have been associated with higher opioid-dose requirements, as they are usually more sensitive to pain, and are more predisposed to compulsive behaviours and opioid dependence compared to rs1799971-A carriers [18,19]. In addition, in this work we have studied the effect of the interaction of G allele–DNA methylation, and the data show that as the *OPRM1* methylation increased, a decrease in the G-allele-reducing MEDD was observed. In line with our result, a previous study on *OPRM1* methylation of 22 CpG sites (including the five selected sites) analyzed 133 adolescents and reported that hypermethylation of the gene leads to a decreased response to opioids with an increased experience of pain [39].

### 4.3. COMT Methylation and Analgesic Response

Our results showed low variability and methylation values close to zero (0.54–1.52%) in the *COMT* promoter region. However, despite the low values, a negative association between pain relief and quality of life was found and patients were more likely to present different adverse events. According to the literature, higher *COMT* expression could increase dopamine degradation in the brain while being more sensitive to pain relief, but different adverse events appeared [40] as in our study. In fact, the *COMT* gene plays a critical role in the synaptic catabolism of neurotransmitters in the prefrontal cortex, where dopamine is crucial and involved in the pharmacological mechanisms of psychostimulant effects [41]. Furthermore, some sex-specific differences have been observed in the response of dopamine neurons in the attenuating pain of female rats [42,43], and in relation to the variability of behavioural and physiological correlates of cognitive control [44,45]. There are accumulating and sometimes compelling data showing that *COMT* has marked sexually dimorphic effects on brain function and its dysfunction in psychiatric disorders [46]. However, our results did not evidence of any sex influence.

Finally, it is well-known that age, sex, psychological status, disabilities, and cultural expectations may influence individual responses to chronic pain [47]. In our study, sex differences were related to significantly older age and higher anxiety levels in females. They should be closely analysed in terms of biopsychosocial mechanisms by adjusting for other confounding factors, such as gender bias due to pain normalisation in females, which may underlie these sex differences [48,49]. Furthermore, females have been described to report being prescribed more anxiolytics, sedatives, or hypnotics which could contribute to OUD [50]. However, our data suggested greater OUD behaviour for males, which agrees with other clinical evidence [51]. All this information needs to undergo a multidimensional approach to assess its impact, plus the epigenetic/genetic influence, on CNCP analgesic response.

### 4.4. Limitations

This study has some limitations that need to be acknowledged. Due to its retrospective design, the data collection of some variables could have been limited by lacking some information reported by clinicians. Additionally, as patients were on concomitant medication to treat other pathologies, unmeasured factors could have contributed to the observed differences. They could have independently contributed to the observed adverse events and differences in pain care [52,53]. The sample size was limited to DNA samples available from a single pain unit but included subjects from different trials, which could add heterogeneity. So, the relatively high OUD incidence in our setting could have affected the results, which need to be replicated in a more diverse population. In addition, it should be noted that some other important factors, such as pain duration, body-mass index, testosterone/estrogen levels, or other lifestyle influences were not controlled in this study. All these factors could introduce a mediated bias that could be more relevant than the pain itself. Therefore, it would be necessary to replicate this analysis, including other factors that could influence our results, in order to reach more accurate conclusions. Nevertheless, one of the strengths of our study lies in the fact that the data was obtained from real-world outpatients. Finally, some analytical limitations have also emerged. We have found evidence of an association between *COMT* gene methylation values and the level of relief and quality of life. Interestingly, the *COMT* promoter site shows methylation values close to zero and with very little variability, so the findings of these analyses should be taken with caution as they may be due to other uncontrolled factors.

## 5. Conclusions

Sex differences in *OPRM1* DNA methylation that impact OUD were proposed and discussed. In addition, we have also found an *OPRM1* genotype/methylation interaction with MEDD, plus an association of *COMT* DNA methylation with pain relief and quality of life in real-world outpatients with CNCP. The study of new factors such as sex and DNA methylation could lead to the identification of new biomarkers to improve analgesic response as a fundamental step towards precision medicine.

## Figures and Tables

**Table 1:** Demographic and clinical data in the total population according to sex. Values are %, mean (standard deviation), or median [interquartile range].

	Total *n* = 250	Females *n* = 125	Males *n* = 125
Age	62 (14)	64 (14) *	59 (14)
Employment status (%)			
At work	10	10	10
Retired	59	68	52
Work Disability	31	22	38
Diagnosis (%)			
Nonspecific low back pain	83	95 **	65
Other pain	17	5	35
Pain intensity (0–100 mm)	67 (21)	68 (22)	66 (20)
Relief (0–100 mm)	32 (27)	34 (26)	30 (28)
Quality of life (0–100 mm)	43 (23)	40 (22)	46 (23)
HAD-Anxiety (0–21 scores)	8 [3, 12]	9 [5, 13] *	5 [2, 11]
HAD-Depression (0–21 scores)	7 [4, 12]	8 [5, 13]	7 [3, 11]
MEDD (mg/day)	106 (99)	104 (99)	109 (98)
Total Adverse Events	3 [1, 6]	3 [1, 6]	3 [1, 5]
Opioid use disorder (%)	21	15	26 *
Adverse Events (%)			
Dry Mouth	45	53	41
Constipation	41	46	42
Insomnia	28	34	26
Dry Skin	22	31 *	16
Nervousness	26	30	26
Dizziness	26	32	23
Sexual disturbance	25	20	33 *
Weight changes	23	33 *	16
Lack of appetite	13	17	11
Red skin	11	27	13

HAD: Hospital Anxiety and Depression Scale; MEDD: Morphine Equivalent Daily Dose. * Denotes *p* < 0.05 and ** denotes *p* < 0.01 when comparing females to males. The highest value is shown in bold.

**Table 2:** DNA Methylation (%) as the mean (standard deviation) at the CpG sites selected in genes *OPRM1* (sites 1–5) and *COMT* (sites 1–7) (counted from the adenine of the start codon).

Code	CpG Sites	Total *n* = 250	Female *n* = 125	Male *n* = 125	*p*-Value
*OPRM1* DNA Methylation (%)					
CpG 1	−32	8.2 (3.8)	8.3 (3.6)	8.1 (4.1)	0.3
CpG 2	−18	16.6 (6.2)	16.6 (5.8)	16.7 (6.7)	0.8
CpG 3	−14	14.2 (5.5)	14.2 (5.0)	14.2 (6.1)	0.5
CpG 4	−10	10.1 (3.9)	10.2 (3.5)	10.0 (4.3)	0.4
CpG 5	+12	8.3 (4.0)	8.3 (3.5)	8.3 (4.5)	0.4
*COMT* DNA Methylation (%)					
CpG 1	−89	1.5 (1.0)	1.5 (0.8)	1.5 (1.1)	0.1
CpG 2	−86	0.9 (1.0)	0.8 (0.6)	0.9 (1.2)	0.1
CpG 3	−84	0.7 (0.9)	0.7 (0.7)	0.7 (1.1)	0.05
CpG 4	−75	1.5 (0.9)	1.5 (0.7)	1.5 (1.1)	0.3
CpG 5	−72	0.8 (0.9)	0.7 (0.6)	0.8 (1.1)	0.06
CpG 6	−67	1.1 (1.5)	1.1 (1.6)	1 (1.4)	0.07
CpG 7	−62	0.5 (0.8)	0.5 (0.5)	0.6 (1.0)	0.1

**Table 3:** Sex differences in the association between *OPRM1* and *COMT* DNA methylation and analgesic response.

	Estimate	SD	*p*-Value
Pain intensity			
*OPRM1*	−0.079	0.250	0.751
*COMT*	0.717	1.114	0.520
Sex	−0.521	3.023	0.863
Relief			
*OPRM1*	0.248	0.294	0.400
*COMT*	−3.149 *	1.344	**0.020**
Sex	3.326	3.65	0.363
Quality of life			
*OPRM1*	0.190	0.238	0.425
*COMT*	−2.069 *	1.028	**0.046**
Sex	−2.108	2.83	0.457
HAD-Anxiety			
*OPRM1*	−0.178 *	0.088	**0.046**
*COMT*	0.228	0.273	0.404
Sex	1.869 *	0.891	**0.039**
HAD-Depression			
*OPRM1*	−0.072	0.081	0.378
*COMT*	−0.011	0.251	0.965
Sex	0.78	0.821	0.345
Opioid Use Disorder			
*OPRM1*	−0.165 **	0.036	**<0.001**
*COMT*	0.018	0.104	0.859
Sex	−2.123 **	0.772	**0.006**
*OPRM1:* Sex	0.099 *	0.05	**0.047**
MEDD (mg/day)			
*OPRM1* G-allele	−0.914 **	0.24	**<0.001**
*OPRM1*	−0.023 **	0.008	**0.005**
*OPRM1*: *OPRM1* G-allele	0.046 **	0.014	**0.001**
Sex	0.009	0.081	0.908

*OPRM1* (CpG2 site), *COMT* (CpG6 site); HAD: Hospital Anxiety and Depression Scale; MEDD: Morphine Equivalent Daily Dose * Denotes *p* < 0.05 and ** denotes *p* < 0.01, *p*-value <0.05 is shown in bold.

**Table 4:** Probability (%) of the DNA methylation effect on the different adverse events.

Adverse Event	Estimate	SD	− Effect Prob.	+ Effect Prob.
Constipation				
*OPRM1*	0.035	0.022	0	25.5
*COMT*	−0.299	0.17	**95.84**	0.36
Insomnia				
*OPRM1*	0.02	0.024	0.37	14.43
*COMT*	−1.145	0.494	**99.92**	0
Dry mouth				
*OPRM1*	0.009	0.022	0.32	2.99
*COMT*	−0.416	0.215	**98.66**	0.08
Dry skin				
*OPRM1*	0.006	0.026	3.66	8.88
*COMT*	−0.648	0.381	**98.16**	0.37
Lack of appetite				
*OPRM1*	−0.03	0.035	42.15	2.37
*COMT*	−1.191	0.636	**98.89**	0.44
Red skin				
*OPRM1*	0.045	0.035	2.2	68.85
*COMT*	−0.765	0.56	**95.24**	2.6
Nervousness				
*OPRM1*	−0.05	0.027	58.74	0.01
*COMT*	−0.341	0.253	**91.11**	2.35
Dizziness				
*OPRM1*	−0.056	0.028	66.16	0
*COMT*	0.211	0.104	0.53	**95.4**

*OPRM1*-CpG2 site, *COMT*-CpG6 site; (−) or (+) Effect probabilities >90% are shown in bold.

## Data Availability

The data presented in this study are available on request from the corresponding author. The data are not publicly available because they contain clinical data of patients.

## Appendix A

**Table A1:** Primers used in the pyrosequencing assay.

Primer	Sequence	CpG sites
*OPRM1*_F1	5′-GGATTGGTTTTTGTAAGAAATAGTAGG-3′	
*OPRM1*_R1	5′-ATACRCCAAAACATCAATACAATTACTAAC-3′	
*OPRM1*_S1	5′-AAGTTTYGGTGTTTTTGGTTA-3′	CpG 7–11
*COMT*_F1	5′-GTGGGGTTTTTGGGGTAGT-3′	
*COMT* _R1	5′-ATCTAACCAACRCTCTCACCTCTCCC-3′	
*COMT* _S1	5′-GGGTTTTTGGGGTAGTTA-3′	CpG 37–42

**Table A2:** Pharmacological data in the total population according to sex.

	Total *n* = 250	Females *n* = 125	Males *n* = 125
Main opioid (%)			
Buprenorphine	5	2	9
Fentanyl	24	26	22
Morphine	8	5	11
Oxycodone	19	25	13
Tapentadol	31	29	33
Tramadol	12	14	11
